# Albany Medical School

**Published:** 1849-02

**Authors:** 


### ALBANY MEDICAL COLLEGE.

We have before us the catalogue and circular of the Albany Medical College, from which we learn that the number of students at the institution in 1848 was 101. A list of the graduates since 1839 is given, numbering 124. The lectures in this school commence on the first Monday in October, and continue sixteen weeks. The fees for a full course amount to $70. The faculty is an able one, and, among the men known to fame, embraces T. Romeyn Beck, author of the popular and learned work on Medical Jurisprudence.

